# #DidacticsRevolution: Applying Kotter’s 8-Step Change Management Model to Residency Didactics

**DOI:** 10.5811/westjem.2019.11.44510

**Published:** 2019-12-19

**Authors:** Mary R. C. Haas, Brendan W. Munzer, Sally A. Santen, Laura R. Hopson, Nathan L. Haas, Daniel Overbeek, William J. Peterson, James A. Cranford, Robert D. Huang

**Affiliations:** * University of Michigan Medical School, Department of Emergency Medicine, Ann Arbor, Michigan; † Virginia Commonwealth University Health System, Department of Emergency Medicine, Richmond, Virginia

## Abstract

**Introduction:**

Leading change effectively is critical to advancing medical education. Residency didactics often require change in order to meet stakeholder’s needs. Kotter’s change management model (KCMM) is an 8-step method for implementing change that can be applied to educational initiatives. This innovation improved an emergency medicine residency didactics curriculum through application of KCMM.

**Methods:**

An initiative to improve residency didactics curriculum was titled the “Didactics Revolution” and implemented according to KCMM: establish a sense of urgency, form a powerful guiding coalition, create a vision, communicate the vision, empower others to act on the vision, plan for and create short-term wins, consolidate improvements and produce still more change, and institutionalize new approaches. Data from the Annual Program Review was utilized to assess the impact of the KCMM strategy.

**Results:**

The percentage of residents who agreed or strongly agreed that lectures provide a valuable learning experience increased from 39.1% in the year prior to 88.0% in the year during the implementation (p < .001), and remained relatively high at 73.5% in the year following. The percentage of residents who agreed or strongly agreed that they felt well-prepared for the written boards increased from 60.9% in the year prior to 92.0% in the year during the implementation (p = .01) and remained high at 73.5% in the year following.

**Conclusion:**

Residency didactics can be improved through the use of KCMM, a change management model originally developed in the corporate context.

## BACKGROUND

While not all changes lead to improvement, all improvement requires change.^1^ As learners’ needs evolve, medical education curricula will necessitate change.^2^ Effective change management is thus critical for the advancement of medical education.^3^

A common curricular area necessitating change and continuous development is residency didactics. Weekly didactics are required by the Accreditation Council for Graduate Medical Education (ACGME) to supplement clinical learning experiences for residents.^4^ However, traditional podium-based, hour-long didactics often fail to engage learners when compared to interactive, shorter educational sessions that encourage active learning.^5^–^10^ Specific changes rooted in education theory that have previously been implemented to improve didactics include shorter, more focused lectures, diverse, interactive teaching formats, and interleaving of both topics and formats.^11^

Implementing changes to didactic curricula can be challenging and requires an approach that engages and meets the needs of various stakeholders. Several models for change have been described, most frequently in the business literature.^12^–^22^ John Kotter, a Harvard Business School Professor and expert on change leadership, designed an 8-step model for leading change.^23^–^24^ Although Kotter’s change management model (KCMM) was originally described in the corporate context, it has been applied previously to human service and educational organizations.^25^–^27^ KCMM incorporates themes that underlie effective change management strategies, including entering and contracting change activities, diagnosing areas for improvement and expansion, planning and implementing, and evaluating and institutionalizing change.^28^ With its simple 8-step approach and ability to engage stakeholders in the change process, KCMM provides a valuable framework for approaching curriculum change within medical education.

## OBJECTIVE

The objective of this innovation was to improve the residency didactics curriculum through application of KCMM.

## METHODS

**Figure 1:**
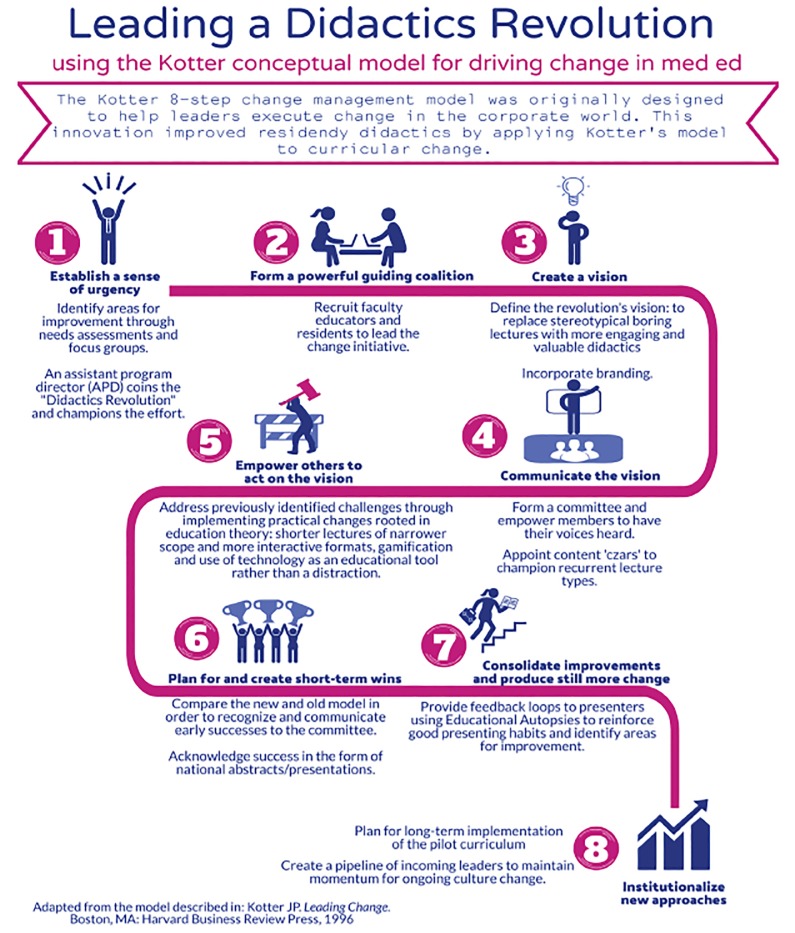
Method of improving EM residency didactics utilizing Kotter’s change management model (KCMM).

KCMM was implemented to improve the didactic curriculum at an academic, four-year emergency medicine (EM) residency program that includes 64 residents and 27 core faculty (Figure 1).

### Establish a Sense of Urgency

An assistant program director (APD) took the lead in recognizing and communicating the need for change. The APD held focus groups with both faculty and residents. The groups identified residents as the key stakeholders and reviewed their perspectives and needs. These discussions confirmed examples of the need for change, including a trend toward attendees sitting in the back of the lecture hall distracted by laptops and smartphones, and the perception that many lectures were too long and overly broad. The lead APD also administered a needs assessment survey with proposed lecture topics and speakers to allow residents to select those of the most interest and value. At the Annual Program Review, the results were shared to promote ongoing discussion and generate buy-in. The change initiative was boldly named the “Didactics Revolution” (DR) and was widely publicized as part of Program Improvement Plans to create excitement and encourage participation. These initiatives increased awareness of existing dissatisfaction with didactics, which drove momentum for change.

### Create a Powerful Guiding Coalition

In order to engage stakeholders, the lead APD created a formal committee called the “Didactics Revolution Committee” (DRC) and recruited specific individuals representing well-respected educators and residents most vocally expressing the desire for change. Five core faculty and 20 EM residents joined. The DRC met monthly after conference to review the current state of the project, provide feedback, brainstorm future developments, and communicate the vision to the rest of the residency. Resident members were selected as “czars” of different lecture types, tasked with ensuring speaker availability, maintaining topic lists, and championing the DR. The program director was aware and supportive of the DRC.

### Create a Strategic Vision

The lead APD and DRC generated the vision of replacing stereotypical boring lectures with more engaging and valuable didactics. Inspiration for specific changes to implement was drawn from the needs assessment survey and focus groups previously mentioned, as well as another EM residency program’s efforts to similarly transform the didactic curriculum.^11^ Initiatives included shortening and narrowing the scope of lectures, creating more interactive and engaging lecture formats, gamifying educational activities, and utilizing social media and technology as educational tools.

### Communicate the Vision

The power of the DRC was harnessed to disseminate the vision of making conference more valuable and engaging. An email about the initiative was distributed to the residency. Members of the DRC made announcements at residency and faculty meetings. Resident “czars” of the DRC communicated the vision to speakers who signed up to deliver lectures, providing guidance for content to cover, lecture duration, slide design and supplemental materials. Branding initiatives included the deliberately chosen title of the “Didactics Revolution” with an associated logo designed to reflect the goal of challenging the status quo.

### Empower Others to Act on the Vision

Changes inspired by the survey, focus groups, and efforts of other residency programs were implemented by removing existing barriers. To support speakers in delivering more engaging lectures, most lectures were shortened from 60 to 15–30 minutes and covered narrower topics through the following series: Visual Diagnosis, electrocardiogram (ECG) of the Week, Rapid Fire Radiology, Case of the Week, and Top 5 Differential Diagnoses.^11^ All residents were encouraged to sit in the front of the auditorium to maximize participation. To transform social media and mobile technology from a distraction into a learning tool, conference content was shared via the residency blog and Twitter account with the hashtag #EMConf. An online, interactive multiple-choice platform entitled Kahoot! incorporated 5 board-review style questions weekly to encourage retention. Lastly, gamification was employed through a knowledge competition entitled “Residency House Cup.”

### Plan for and Create Short Term Wins

The DRC seized and publicized opportunities for success during the early implementation process. New content was developed alongside old content. At monthly meetings, the DRC compared the two models to recognize improvements and gain confidence to try additional innovations. Resident and faculty champions were rewarded for success through the acceptance of presentations detailing the initiative at national meetings. A poster presentation entitled **“**An Emergency Medicine Residency Didactics Revolution: The Use of a Multidisciplinary Team and Branding to Inspire and Support Curricular Change” was presented at the 2017 Council of Emergency Medicine Residency Directors (CORD) Academic Assembly. A component of the DR called “Educational Autopsy” was featured in a poster presentation entitled “Highlighting Themes in Emergency Medicine Didactics Using the Educational Autopsy” at the 2017 Society for Academic Medicine (SAEM) Annual Meeting.

### Consolidate Improvements and Produce Still More Change

Feedback was sought and continuously incorporated to push for ongoing improvement. The DRC implemented “Educational Autopsy” (EA), a 30-minute session run by a member of the residency leadership following each conference day. During EA, conference attendees dissected each presentation for strengths and weaknesses and assessed whether it reflected the strategic vision. Feedback was emailed to individual speakers. General themes were reviewed at each DRC meeting. This resulted in the addition of new sessions, removal of old sessions, and the implementation of lessons learned from others by DRC members developing future presentations.

### Institutionalize New Approaches

The infrastructure for ongoing progress toward meeting the strategic vision was implemented by developing a two-year conference curriculum based on lessons learned during the initial pilot year. The state of didactics remains in a continuous process of reevaluation and improvement. New leaders are identified within the younger classes and encouraged to become more involved in content creation, promoting sustainability and institutionalization of the curricular change.

## IMPACT/EFFECTIVENESS

Yearly, the residency participates in an annual program review process, which includes a survey administered to all residents and faculty. Data from the annual program review survey were collected for the academic years preceding (2015–2016, n = 23, response rate = 36.5%), during (2016–2017, n = 25, response rate = 39.7%), and after (2017–2018, n = 34, response rate = 53.1%) a year of implementation of the DR. The Institutional Review Board determined that the use of this data for research was exempt. Residents evaluated six items (e.g., “Lectures provide valuable learning experience”) on a scale of 1 = strongly agree to 5 = strongly disagree. To facilitate interpretation, percentage of residents who responded “strongly agree” or “agree” to each item was compared across all three years.

The percentage of residents who agreed or strongly agreed that a) small group sessions provide valuable learning experiences; b) simulation sessions provide valuable learning experiences; and c) they were confident in their ability to critically appraise the medical literature was relatively high in the year prior to DR, and did not show any statistically significant changes in the year during or in the year following DR implementation. By contrast, the percentage of residents who agreed or strongly agreed that lectures provide a valuable learning experience increased from 39.1% in the year prior to DR to 88.0% in the year during DR implementation (χ^1^ [1] = 12.3, *p* < .001, absolute benefit increase = 48.9), and remained relatively high at 73.5% in the year following the DR (χ^1^ [1] = 6.6, *p* = .01, absolute benefit increase = 34.4). In addition, the percentage of residents who agreed or strongly agreed that they would be well-prepared for the written boards increased from 60.9% in the year prior to DR to 92.0% in the year during DR implementation (χ^1^ [1] = 6.3, *p* = .01, absolute benefit increase = 31.1) and remained relatively high at 73.5% in the year following the DR (χ^1^ [1] = 1.0, *p* = .36). Finally, the percentage of residents who agreed or strongly agreed that they would be well-prepared for the oral boards increased from 56.5% in the year prior to the DR to 80.0% in the year during DR implementation and dropped to 61.8% in the year following the DR, but these changes were statistically nonsignificant. See Table 1.

Limitations of the data include relatively low response rates, and that the survey was not specifically designed to assess the impact of the application of KCMM to residency didactics. Nonetheless, results suggest that the didactic curriculum was more engaging and effective following the change initiative. While learner opinion about curricular effectiveness is useful, future study should investigate if KCMM results in improved learning outcomes, such as in training exam scores or medical knowledge milestones. Assessing for an increase in conference attendance was considered as another potential marker of increased engagement, but not utilized given the 70% minimum attendance rate that provides a natural ceiling.

The DR initiative focused primarily on improving didactic experiences specifically, which may explain why no statistically significant changes were identified for learner perceptions about degree of preparation for oral boards, ability to appraise medical literature (which may correlate to quality of journal club), or the value of simulation and small group learning experiences. Assessing impact of change management initiatives targeted toward these educational components represents an area of future study. Additionally, with three of the four items that did not show statistically significant increases, level of agreement was relatively high in the year prior to DR. This suggests a ceiling effect whereby there was less room for change. By contrast, levels of agreement with the two items that showed statistically significant increases were relatively lower in the year prior to DR, allowing more room for improvement.

The use of KCMM for improving the didactic curriculum had several advantages. It provided an easy, step-by-step guide for leaders to approach an area of weakness. It engaged learners in the process of improving their own educational experience. It also encouraged stakeholders to embrace, rather than fear, change. Challenges included the substantial time commitment required of busy residents and faculty to implement this intensive approach. The authors also noted a drop off in improved learner perceptions from the year during the DR to the year following. This may reflect the difficulty in sustaining momentum of change initiatives, where excitement often starts out high but requires significant and ongoing dedication, time, and effort to maintain.

Although originally developed in the corporate context, KCMM provides a valuable framework for leading change in medical education. KCMM can be applied at other programs to restructure didactics or other curricular areas.

## Figures and Tables

**Table 1:** Percentage of residents who responded “Agree” or “Strongly Agree” by item.

Year	2015 (Prior)	2016 (During)	2017 (After)
Survey response rate	36.5%	39.7%	53.1%

Q1. Small group sessions provide valuable learning experiences.	82.6%	88.0%	82.4%
Q2. Simulation sessions provide valuable learning experiences.	100.0%	96.0%	91.2%
Q3. Lectures provide valuable learning experience.	39.1%	88.0%	73.5%
Q4. I am confident in my ability to critically appraise the medical literature.	73.9%	80.0%	79.4%
Q5. I feel that I will be well prepared for the written boards.	60.9%	92.0%	73.5%
Q6. I feel that I will be well prepared for the oral boards.	56.5%	80.0%	61.8%
